# Supplementary material for: Assessment of ultra processed foods consumption in Senegal: validation of the Nova-UPF screener
Source: Arch Public Health. 2024 Jan 10;82:4. doi: 10.1186/s13690-024-01239-y (PMC10777632; doi:10.1186/s13690-024-01239-y)
Supplement: Supplementary file 1 — Supplementary Material 1: Table 1: Dietary share of ultra-processed foods calculated by the 24-hour dietary recall according to the Nova score. Senegal (n = 301), 2021. Table 2: Distribution of Men (%) by UPF energy contribution quintiles (from 24-hour dietary recall) and Nova-UPF score quintiles. Table 3: Distribution of Women (%) by UPF energy contribution quintiles (from 24-hour dietary recall) and Nova-UPF score quintiles [file 13690_2024_1239_MOESM1_ESM.docx]

Table 1: Dietary share of ultra-processed foods calculated by the 24-hour dietary recall according to the Nova score. Senegal (n = 301), 2021.

| **Score quintiles** | **Sample**  **n (%)** | **Mean dietary share of UPF**  **mean (95% CI)** |
| --- | --- | --- |
| 1 | 62 (20.6) | 2.8 (1.5 – 4.2) |
| 2 | 82 (27.2) | 14.6 (11.1 – 18.1) |
| 3 | 86 (28.6) | 21.4 (17.8 – 25) |
| 4 | 40 (13.3) | 27.1 (21.8 – 32.5) |
| 5 | 31 (10.3) | 30.4 (24 – 36.7) ^a^ |

^95% CI = 95% Confidence Interval; a = P < 0.001^

Table 2: Distribution of **Men** (%) by UPF energy contribution quintiles (from 24-hour dietary recall) and Nova-UPF score quintiles.

| **Quintiles of energy intake (%) from UPF (full 24h recall)** | **Nova-UPF score quintiles** | | | | | |
| --- | --- | --- | --- | --- | --- | --- |
|  | **1** | **2** | **3** | **4** | **5** | **Total** |
| Q1 (≤ 2.05) | 15.89 | 5.96 | 0.66 | 0.00 | 0.66 | 23.18 |
| Q2 (2.06 – 10.78) | 1.32 | 14.57 | 5.30 | 3.97 | 0.00 | 25.17 |
| Q3 (10.79 – 26.80) | 1.32 | 7.95 | 8.61 | 5.96 | 5.30 | 29.14 |
| Q4 (26.81 – 41.35) | 0.00 | 1.99 | 5.96 | 2.65 | 2.65 | 13.25 |
| Q5 (≥ 41.36) | 0.00 | 1.99 | 0.66 | 3.97 | 2.65 | 9.27 |
| Total | 18.54 | 32.45 | 21.19 | 16.56 | 11.26 | 100 |

^Pabak index (Kappa adjusted for prevalence bias) = 0.85^

Table 3: Distribution of **Women** (%) by UPF energy contribution quintiles (from 24-hour dietary recall) and Nova-UPF score quintiles.

| **Quintiles of energy intake (%) from UPF (full 24h recall)** | **Nova-UPF score quintiles** | | | | | |
| --- | --- | --- | --- | --- | --- | --- |
|  | **1** | **2** | **3** | **4** | **5** | **Total** |
| Q1 (≤ 2.18) | 14.67 | 2.67 | 0.00 | 0.67 | 0.00 | 18.00 |
| Q2 (2.19 – 10.89) | 6.00 | 9.33 | 12.67 | 0.67 | 0.67 | 29.33 |
| Q3 (10.90 – 25.64) | 1.33 | 5.33 | 13.33 | 4.00 | 4.00 | 28.00 |
| Q4 (25.65 – 40.99) | 0.67 | 1.33 | 6.00 | 3.33 | 2.00 | 13.33 |
| Q5 (≥ 41.00) | 0.00 | 3.33 | 4.00 | 1.33 | 2.67 | 11.33 |
| Total | 22.67 | 22.00 | 36.00 | 10.00 | 9.33 | 100 |

^Pabak index (Kappa adjusted for prevalence bias) = 0.83^
